# The temporal sequence of influenza H1N1 and *Mycoplasma pneumoniae* co-infection causes disease severity in Syrian hamster models

**DOI:** 10.3389/fmicb.2026.1787294

**Published:** 2026-03-27

**Authors:** Junchi Ma, Yanyan Li, Weihua Jin, Xueqian Xie, Aiyi Li, Yanan Wu, Fengmei Yang, Suqin Duan, Mingxue Li, Haiting Long, Zhanlong He, Yuan Zhao

**Affiliations:** 1 Institute of Medical Biology, Chinese Academy of Medical Sciences and Peking Union Medical College, Kunming, China; 2 College of Life Sciences, Yunnan University, Kunming, China

**Keywords:** co-infection, influenza H1N1 virus, *Mycoplasma pneumoniae*, respiratory infectious disease, Syrian hamster, temporal sequence

## Abstract

**Introduction:**

Influenza H1N1 virus is one of the most prevalent subtypes among influenza viruses, and co-infection with *Mycoplasma pneumoniae* (Mp) is frequently documented in clinical respiratory infections. However, the pathological mechanisms underlying the temporal sequence of H1N1-Mp co-infection remain poorly characterized, and relevant animal models are lacking.

**Methods:**

In this study, we established a model of influenza H1N1 and *Mycoplasma pneumoniae* co-infection in Syrian hamsters and infected two pathogens in interval of 72 hours. Clinical manifestations, body temperature, body weight, pathogen loads in nasal, pharyngeal, and anal swabs, as well as blood cytokine profiles were dynamically monitored over 14 days post-infection (dpi). Additionally, tissue pathogen loads, histopathological changes, routine blood parameters, and blood biochemistry indicators were evaluated at 7 and 14 dpi.

**Results:**

The results demonstrated that hamsters first infected with H1N1 followed by Mp (F-M group) exhibited significantly more severe histopathological lesions (assessed by HE staining), higher pathogen loads, and dysregulated cytokine responses compared to other infection groups.

**Conclusion:**

Our findings highlight the critical role of infection order in determining the severity of H1N1-Mp co-infection, providing novel insights into the temporal dynamics and pathogenic mechanisms of respiratory co-infections.

## 1 Introduction

Respiratory viral-bacterial co-infections represent a significant global health burden, contributing to increased morbidity and mortality, particularly in vulnerable populations such as children, the elderly, and immunocompromised individuals (Morens and Fauci, 2007; Murray et al., 2006; Zhong et al., 2003). Seasonal influenza A virus (IAV) infections alone cause millions of severe cases annually (Bridges et al., 2003; Clohisey and Baillie, 2019; Krammer et al., 2018; Morens and Taubenberger, 2019; Nakagawa et al., 2017; Svyatchenko et al., 2023), while *Mycoplasma pneumoniae* (Mp), a common atypical bacterial pathogen, often coexists with viruses in acute respiratory illnesses (Nour et al., 2005; Yamanaka et al., 2012). Accumulating evidence indicates that co-infections with IAV and Mp result in more severe disease outcomes than single-pathogen infections (Li et al., 2021). However, the temporal dynamics governing their synergistic interactions remain incompletely understood, which hinders the development of targeted therapeutic strategies and underscores the need for mechanistic studies in relevant animal models (Li et al., 2021; Xu et al., 2024).

Influenza viruses, including the H1N1 subtype, disrupt the respiratory epithelial barrier through neuraminidase-mediated damage, impair mucociliary clearance, and modulate host immune responses—creating a permissive microenvironment for secondary bacterial colonization (Krammer et al., 2018; Herold et al., 2015). Similarly, Mp adheres to the respiratory epithelium cells via the P1 adhesin, inducing chronic inflammation through community-acquired respiratory distress syndrome (CARDS) toxin and dysregulates cytokine networks (Atkinson et al., 2008). Clinical studies have reported elevated hospitalization rates and mortality in patients with IAV-Mp co-infections (Cillóniz et al., 2012; Lalbiaktluangi et al., 2023; Ruuskanen et al., 2011).

Animal models are essential for dissecting the complex interactions between pathogens and the host. Syrian hamsters have emerged as a robust surrogate for human respiratory infections due to their physiological similarity to humans in viral replication kinetics, clinical symptomology, and immune response patterns (Cimolai et al., 1995; Cimolai et al., 1992; Iwatsuki-Horimoto et al., 2018). Previous studies using hamsters have demonstrated IAV-induced pulmonary pathology and Mp-mediated airway inflammation (Iwatsuki-Horimoto et al., 2018; Waites et al., 2017; Zhang et al., 2021), but few have systematically evaluated how the sequence of pathogen exposure influences disease outcomes. Most existing research focuses on simultaneous co-infection or post-viral bacterial superinfection, leaving the impact of temporal spacing and sequence largely uncharacterized (Lalbiaktluangi et al., 2023).

Despite advances in understanding respiratory co-infections, several key questions remain unresolved. First, while murine models predominate in preclinical research, golden hamsters have been extensively employed in the study of respiratory infectious diseases. Therefore, we aimed to explore their potential utility in modeling respiratory infectious disease co-infections (Nguyen et al., 2021; Zhang et al., 2021; Lee et al., 2020). Second, the role of the temporal sequence of infection—specifically, whether prior exposure to IAV enhances Mp pathogenicity or vice versa—has not been rigorously investigated. Third, the immunological mechanisms driving differential severity in sequential co-infections, such as cytokine storm dynamics and tissue tropism, require further elucidation. These gaps limit translational insights into clinical management, where the timing of diagnosis and intervention could critically influence patient outcomes.

Previous studies of influenza–bacterial coinfection have predominantly focused on classical pathogens such as *Streptococcus pneumoniae* and *Staphylococcus aureus*, largely using murine models (Lalbiaktluangi et al., 2023; Nakamura et al., 2011; Chertow and Memoli, 2013). In contrast, coinfection involving *Mycoplasma pneumoniae* and influenza virus remains relatively underexplored despite its clinical relevance. Moreover, coinfection is often treated as a static condition, whereas the temporal sequence of infection may critically influence host immune responses, pathogen dynamics, and disease severity (Lalbiaktluangi et al., 2023). Incorporating infection order as an experimental variable may therefore provide a more mechanistic understanding of host–pathogen interactions during sequential respiratory infections.

This study aims to establish a Syrian hamster model to investigate the impact of temporal sequence in H1N1 and Mp co-infection on disease pathogenesis. By comparing sequential infection (H1N1 followed by Mp, and Mp followed by H1N1), simultaneous co-infection, and single-pathogen infection, we systematically assessed changes in clinical manifestations, pathogen replication kinetics, and tissue pathology. Clinical studies have shown that secondary bacterial infections following influenza typically occur within 3–7 days after the onset of flu symptoms. This window corresponds to the period of most severe epithelial damage and dysregulated innate immune responses (McCullers, 2006; Morens and Fauci, 2007; Bakaletz, 2017). Therefore, we selected a 72-h interval between challenges with the two pathogens. Further analysis of cytokine profiles and immune markers (such as leukocytes, CRP, and complement proteins) associated with severe outcomes was conducted to characterize the immune response. This work validates the utility of the hamster model for studying the dynamics of human-relevant co-infections.

This manuscript presents the methodology, results, and discussion of our investigation into the temporal sequence of H1N1-Mp co-infection. Section 2 describes the experimental design, including animal ethics, pathogen strains, and analytical approaches for pathogen quantification and histopathology. Section 3 details the clinical, immunological, and pathological findings, highlighting the enhanced severity of H1N1-first co-infections. Section 4 discusses the mechanistic implications of these results, contextualizes them within existing literature, and addresses the model’s translational relevance. Together, these findings provide critical insights into the temporal determinants of respiratory co-infections and inform future therapeutic development.

## 2 Materials and methods

### 2.1 Ethical statement

All experimental procedures involving Syrian hamsters conducted in this study were in strict compliance with the Animal Ethics Protocols of the People’s Republic of China and approved by the Animal Welfare Ethics Committee of the Institute of Medical Biology, Chinese Academy of Medical Sciences [Ethical review number: DWSP202409010]. The study adhered to the 3R principles (Replacement, Reduction, and Refinement) and the guidelines of the National Institutes of Health (NIH) and the International Council for Laboratory Animal Science (ICLAS).

### 2.2 Animals

Six-week-old female specific pathogen-free (SPF) Syrian hamsters with defined genetic backgrounds were purchased from Beijing Vital River Laboratory Animal Technology Co. Ltd. [License number: SCXK (Beijing) 2021-0011]. Antibody testing confirmed the absence of antibodies against influenza A virus and Mp. Hamsters were housed in the animal facility of the Department of Laboratory Animals, Institute of Medical Biology, Chinese Academy of Medical Sciences [License number: SYXK (Yunnan) K2021-0001]. All experimental procedures were conducted in the ABSL-2 laboratory at the Institute of Medical Biology, Chinese Academy of Medical Sciences (Approval number: 2024SW0004), with animals having free access to food and water.

### 2.3 Pathogen strains

*Mycoplasma pneumoniae* (ATCC 19342/M129 strain) was kindly provided by Professor Guoyang Liao (Institute of Medical Biology, Chinese Academy of Medical Sciences), and influenza A virus strain A/Puerto Rico/8/34 (H1N1, PR8) was gifted by Professor Shuaiyao Lu (Institute of Medical Biology, Chinese Academy of Medical Sciences). Both strains were stored in their respective laboratories.

### 2.4 Experimental strategy

**Figure 1:**
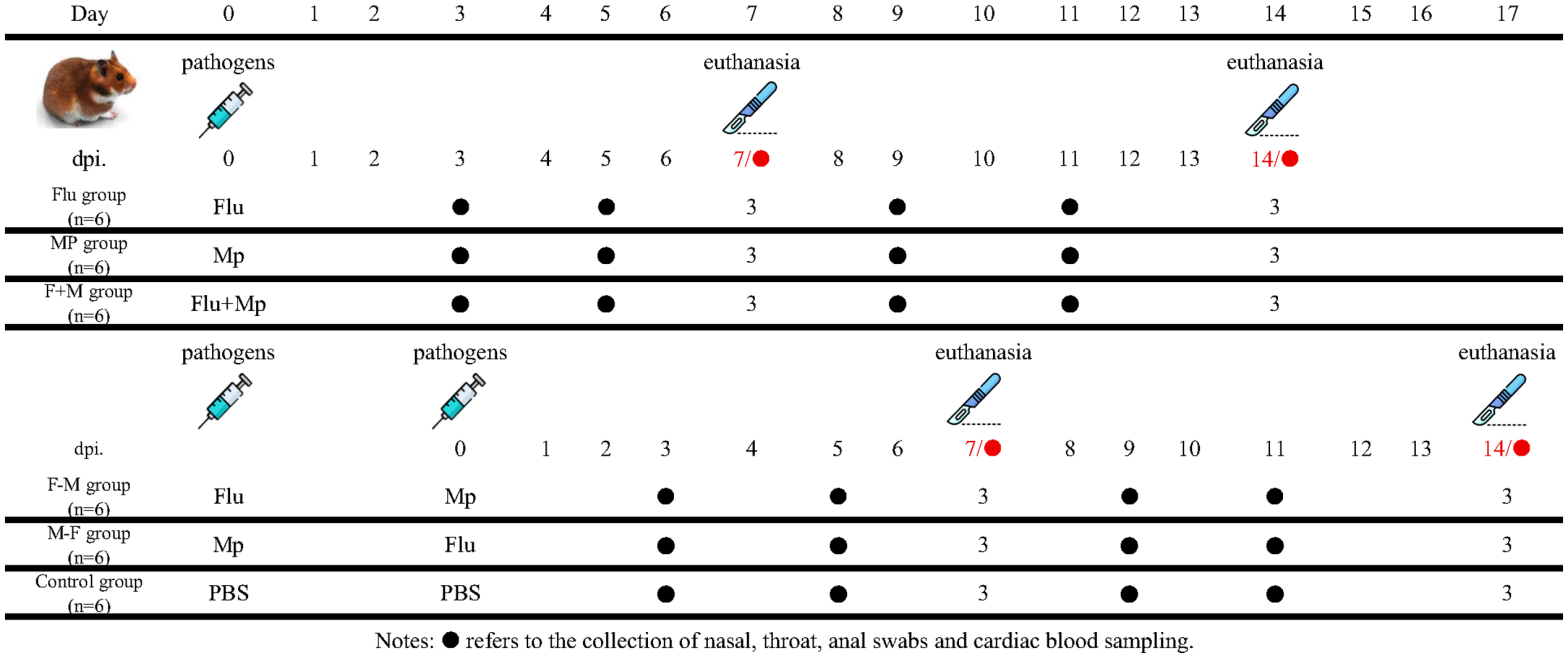
The experiment design of Syrian hamster co-infection.

Thirty-six Syrian hamsters were randomly divided into 6 groups (n = 6 per group): control group (C group, PBS treatment), H1N1 single-infection group (Flu group), Mp single-infection group (MP group), simultaneous H1N1-Mp co-infection group (F+M group), infection with Mp followed by H1N1 infection (M-F group, 72-h interval), and H1N1 followed by Mp infection (F-M group, 72-h interval). For infection, each hamster received 100 μL of H1N1 (10^7.5^ TCID_50_/mL) via intranasal drip (Bouvier and Lowen, 2010; Nguyen et al., 2021). Mp infection was performed with 100 μL of Mp (1 × 10^7^ CCU/mL) via tracheal injection combined with intranasal drip (Dumke et al., 2004; Xie et al., 2025). The control group was treated with an equivalent volume of PBS buffer solution (Figure 1). Specifically, 0 dpi was defined as the time point when the inoculation of both pathogens had been completed. At 7 and 14 dpi in each group, three animals were randomly selected and anesthetized with ketamine at a dose of 300 mg/kg, followed by dissection.

Hamsters were acclimatized for 7 days prior to infection, and baseline data were collected at 0 dpi. After infection, clinical symptoms (mental state, nasal mucosal changes, behavior) were observed daily. We established a clinical scoring system: three clinical veterinarians independently scored the symptoms and deducted certain points based on observed clinical manifestations. Lower scores indicating more critical conditions. Deduction criteria:

**Table 1:** 

Scoring range	Symptom description
0	Normal
0–1	Reduced activity
0–2	Decreased food intake
0–3	Lethargy and wrinkled fur
0–4	Emaciation and abnormal feces
0–5	Hunched posture, depression, and ataxia
0–6	Coughing, sneezing, ocular mucus discharge, nose blood and lacrimation
0–7	Dyspnea, open-mouth breathing, and cyanosis
0–8	Limb paralysis and convulsions
9–10	Moribund or deceased

Body temperature (abdominal measurement) and weight were recorded at 1, 3, 5, 7, 9, 11, and 14 dpi. Nasal, pharyngeal, and anal swabs were collected at 3, 5, 7, 9, 11, and 14 dpi for pathogen nucleic acid load detection (swab samples were collected at 0 dpi and used as an essential negative control in the experiment). Blood samples were collected at the same time points to measure cytokine levels and pathogen load. Additionally, routine blood parameters and blood biochemistry: white blood cell count (WBC), platelet count (PLT), neutrophil percentage (NEUT%), lymphocyte percentage (LYMPH%) and CRP were analyzed at 7 and 14 dpi. At 7 and 14 dpi, three hamsters per were euthanized, and tissue samples (hilar lymph node, trachea, lung, nasal turbinate, heart, liver, spleen, kidney, stomach, pancreas, jejunum, uterus, olfactory bulb, brain, lung lavage fluid, and nasal lavage fluid) were collected for pathogen load analysis, Hematoxylin and Eosin (H&E) staining and immunofluorescence (IF) assay.

### 2.5 Pathogen load quantification

Total RNA/DNA was extracted from nasal swabs, throat swabs, anal swabs, blood and tissues. H1N1 load was quantified by one-step qRT–PCR using primers and probes recommended in the National Influenza Surveillance Technical Guidelines (2017 Edition). Mp load was determined by qPCR targeting the conserved P1a structural domain (GenBank accession no. M18639.1) (Supplementary Table S1). Based on previous reports, influenza virus can also infect the intestinal tract and cause intestinal damage. Therefore, we have included the detection of pathogen load in anal swabs (Wang et al., 2014). qRT-PCR and qPCR were performed using the CFX96 Touch™ Real-Time PCR Detection System (Bio-Rad Laboratories) with One Step PrimeScript™ RT-PCR Kit (Takara, RR064A) and Premix Ex Taq™ (Probe qPCR) (Takara, RR390A), respectively. The reaction conditions for qRT-PCR were: 42 °C for 5 min, 95 °C for 10 s, followed by 40 cycles of 95 °C for 5 s and 60 °C for 30 s. For qPCR: 95 °C for 30 s, followed by 40 cycles of 95 °C for 5 s and 60 °C for 30 s. Pathogen loads were calculated based on standard curves.

### 2.6 Cytokine assay

Total RNA was extracted from anticoagulated blood, and cytokine mRNA levels (IL-1β, IL-2, IL-4, IL-5, IL-6, IL-10, IL-17a, TNF, IFN-γ) were quantified by qRT-PCR using PPIA and HPRT1 as reference genes (Supplementary Table S2) (Rajkhowa et al., 2024; Tatsumi et al., 2008) qRT-PCR was performed with the One Step SYBR PrimeScript™ RT-PCR Kit (Takara, RR096A) under the following conditions: 42 °C for 5 min, 95 °C for 10 s, followed by 40 cycles of 95 °C for 5 s and 60 °C for 30 s. After the experiment, we adopted a relative quantitative calculation method, using the difference in Ct values between the target gene and the reference gene as the Δ Ct value. Then, based on the difference in Δ Ct values between the experimental group and the control group, we calculated the relative expression level.

### 2.7 H&E staining and IF assay

Tissues were fixed in 4% PFA, dehydrated with gradient ethanol, cleared with xylene, and embedded in paraffin. Sections (2 μm) were deparaffinized, rehydrated, and stained with HE for histopathological analysis. For IF assay, sections were gently boiled in citrate buffer (pH 6.0) for antigen retrieval, blocked with 4% BSA, and incubated overnight at 4 °C with primary antibodies: anti-Mp antibody (Novus, NB100-65504, 1:200) and anti-influenza A M2 monoclonal antibody (Invitrogen, MA1-082, 1:1000). After washing, sections were incubated with DyLight 488-conjugated goat anti-rabbit IgG (Novus, NB7156G, 1:200) and DyLight 650-conjugated goat anti-mouse IgG (Novus, NB7535C, 1:200) for 1 h at 37 °C. Nuclei were counterstained with DAPI (Abcam, ab104139), and images were captured using a fluorescence microscope.

### 2.8 Statistical analysis

GraphPad Prism 10.1.2 was used for statistical analyses, and results are presented as mean ± standard deviation (*x* ± *s*). Differences between groups were analyzed by Student’s *t*-test, with *p* < 0.05 considered statistically significant. Pearson’s correlation analysis was performed using SPSS 27.0.1 to assess relationships between blood parameters, pathogen loads, and cytokine levels and Student’s *t*-test was employed to demonstrate the significance of correlations between groups, with a significance level set at *p* < 0.05.

## 3 Results

### 3.1 Clinical manifestations in infected Syrian hamsters

**Figure 2:**
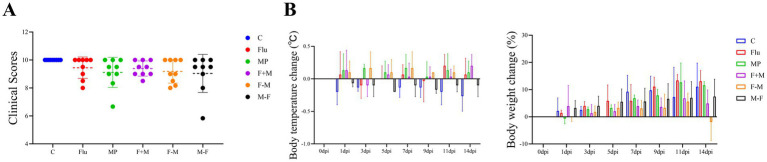
**(A)** Clinical record scoring form of Syrian hamster after infection. **(B)** Body temperature and weight changes monitored in six groups (*n* = 6 per group).

From 3 dpi onwards, all infected groups gradually developed clinical signs such as nasal discharge and nasopharyngeal secretions. The Flu group showed resolution of clinical symptoms by 9 dpi. Nasal bleeding and cyanosis (bluish discoloration of the lips) were observed in the M-F group at 7 dpi, the F+M group at 9 dpi, and the MP group at 11 dpi (Figure 2A). Among these, the MP group demonstrated the most severe symptoms with the longest duration. No significant trend in body temperature changes was observed across all groups (Figure 2B), and overall body weight in each group showed a stable increasing trend (Figure 2B). Notably, temperature changes in the F-M and F+M groups were slightly higher than in the other groups, and the weight of the F-M group slightly decreased on the last day, while the weight increase in the F+M and M-F groups was less pronounced than in the C group.

**Figure 3:**
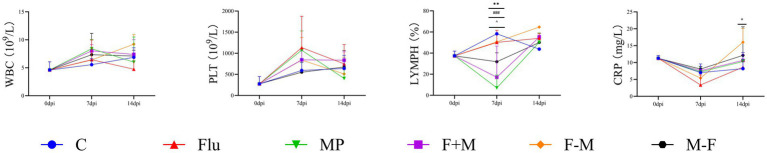
Blood indicators of the coinfection group. ** indicates a significant difference between the F+M group and the F-M group (*p* < 0.01), ### indicates a significant difference between the MP group and the F-M group (*p* < 0.001), ^ indicates a significant difference between the MP group and the M-F group (*p* < 0.05), + indicates a significant difference between the Flu group and the F-M group (*p* < 0.05).

For WBC, at 7 dpi, all infected groups showed higher levels than Group C, with the MP and F+M groups being slightly higher. At 14 dpi, levels in all other groups were similar, except for the Flu group. For PLT, at 7 dpi, all infected groups had higher counts than Group C, with the Flu and MP groups being slightly higher. By 14 dpi, all groups showed a significant decrease. For LYMPH%, at 7 dpi, Group C had the highest level. By 14 dpi, all groups except Group C showed a decrease. For CRP, at 7 dpi, Group C was at a relatively high level, with no substantial differences among groups. At 14 dpi, Group C remained largely unchanged, while all other groups showed increased levels, with the F-M group exhibiting the most pronounced rise (Figure 3).

### 3.2 Influenza H1N1 and *Mycoplasma pneumoniae* replication in Syrian hamsters

**Figure 4:**
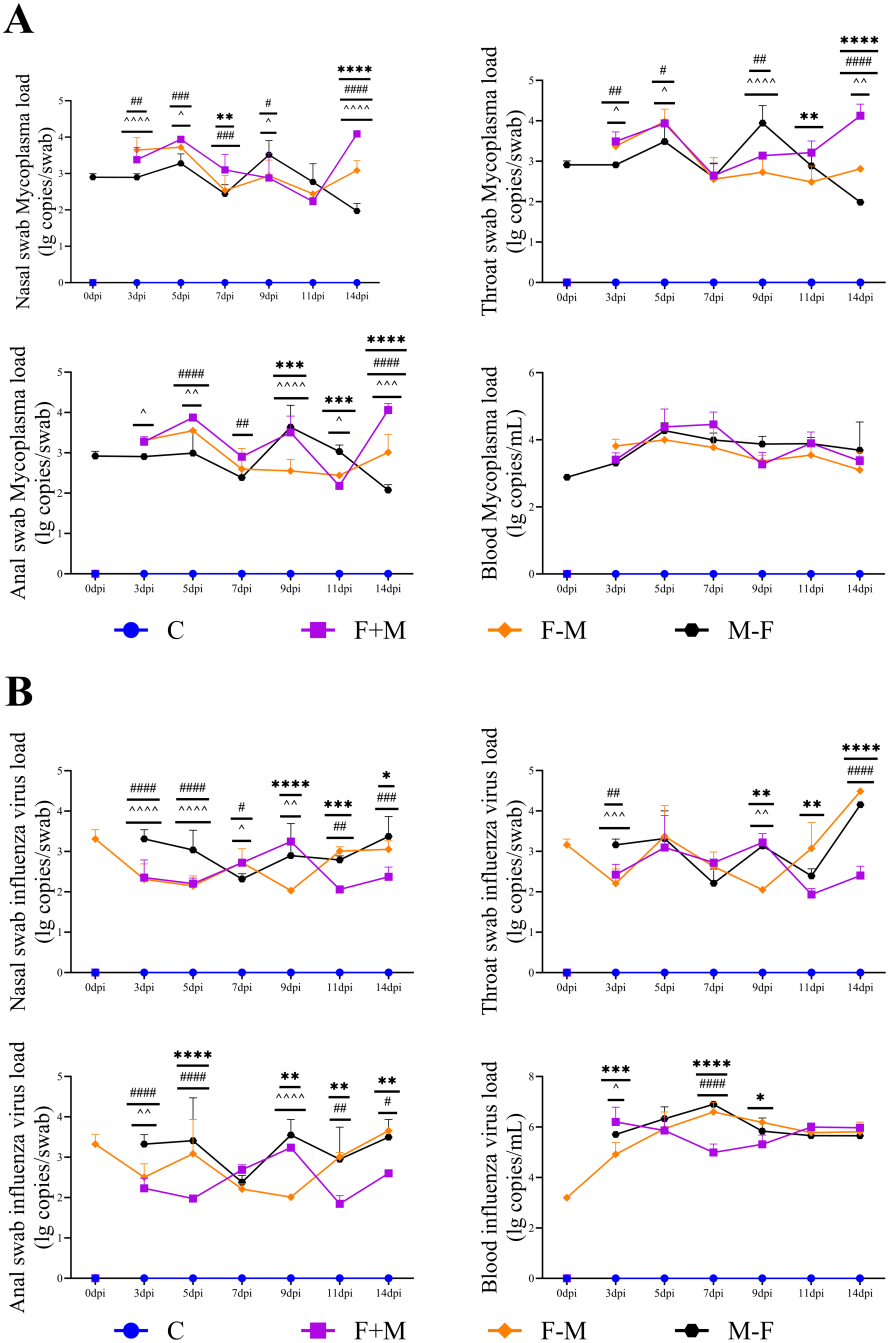
The viral load of the H1N1 influenza virus and the *Mycoplasma pneumoniae* load in nasal swabs, throat swabs, anal swabs, and anticoagulated blood **(A)** *Mycoplasma pneumoniae* load and **(B)** influenza virus load. * indicates a significant difference between the F+M group and the F-M group (*p < 0.05, **p < 0.01, ***p < 0.001, ****p < 0.0001). # indicates a significant difference between the F+M group and the M-F group (#*p* < 0.05, ##*p* < 0.01, ###*p* < 0.001, ####*p* < 0.0001). ^ indicates a significant difference between the F-M group and the M-F group (^*p* < 0.05, ^^*p* < 0.01, ^^^*p* < 0.001, ^^^^*p* < 0.0001).

**Figure 5:**
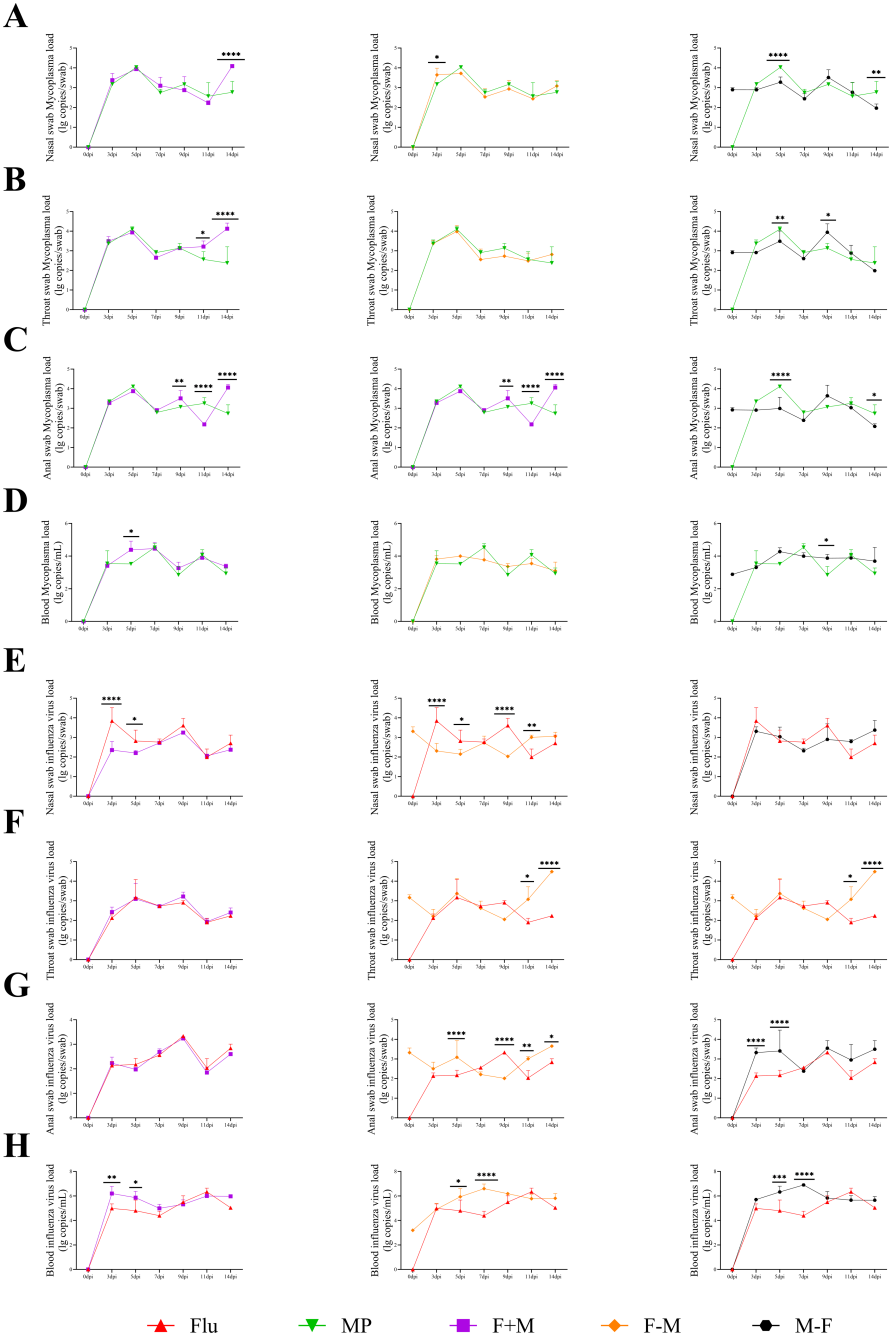
Significant differences in swab pathogen loads between the three coinfection groups and the Flu or MP single-infection groups. **(A–D)** Significant differences for MP load in nasal, pharyngeal, anal swabs, and blood. **(E–H)** Significant differences for influenza viral load in nasal, pharyngeal, anal swabs, and blood (*p < 0.05, **p < 0.01, ***p < 0.001).

We measured pathogen loads in nasal swabs, throat swabs, anal swabs, and blood samples of golden hamsters from 0 to 14 dpi. Pathogens were first detected in swabs at 3 dpi, with most reaching peak loads at 5 dpi followed by a decline. The F+M group showed a rebound in pathogen load at 9 or 14 dpi, while the F-M group (H1N1 load) rebounded at 14 dpi. H1N1 and Mp were detected in nasal, pharyngeal, and anal swabs, as well as blood, throughout the 14-day observation period. These findings provide critical insights into how the virus impacts the body and progresses in this animal model (Figures 4, 5).

**Figure 6:**
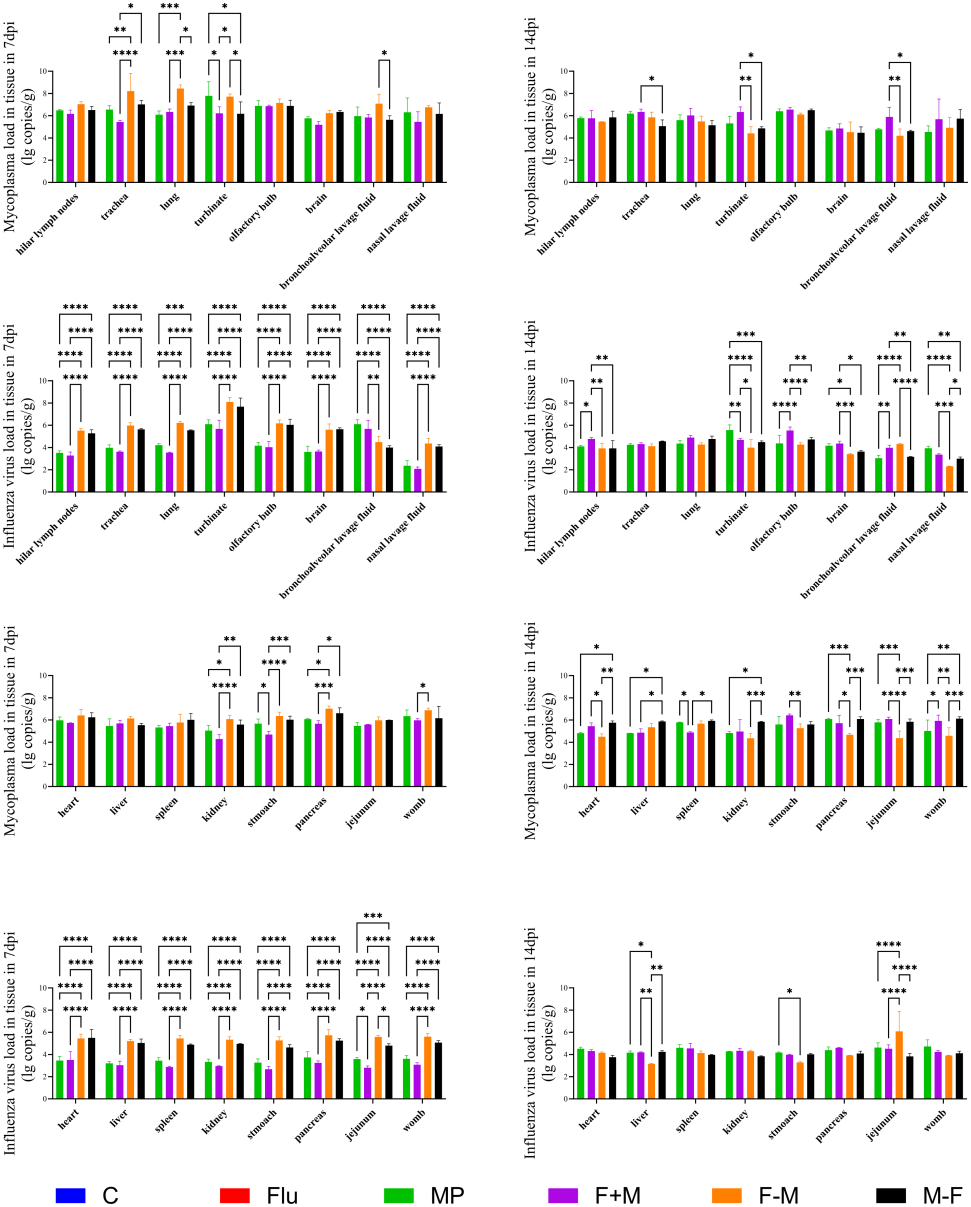
The viral load of the H1N1 influenza virus and the *Mycoplasma pneumoniae* load in relevant tissues (*p < 0.05, **p < 0.01, ***p < 0.001, ****p < 0.0001).

Regarding Mp load across tissues, higher loads were observed in respiratory-related tissues such as lungs, trachea, turbinate, olfactory bulb, bronchoalveolar lavage fluid, and nasal lavage fluid. The overall Mp load at 14 dpi was lower than at 7 dpi. At 7 dpi, the F-M group exhibited higher loads in most tissues compared to other groups, whereas at 14 dpi, the F+M group showed higher loads in the majority of tissues. For influenza virus load in tissues, elevated levels were detected in respiratory-associated tissues including lungs, hilar lymph nodes, trachea, turbinate, and olfactory bulb, but lower levels were found in bronchoalveolar and nasal lavage fluids. Similar to Mp results, the overall influenza virus load at 14 dpi was reduced compared to 7 dpi. At 7 dpi, the F-M group had higher viral loads in most tissues relative to other groups, while at 14 dpi, the F+M group demonstrated higher loads in most tissues. At 7 dpi, in respiratory-related tissues, the three co-infection groups exhibited higher viral and Mp loads compared to single-pathogen infection groups. At 14 dpi, except in the trachea, the three co-infection groups also showed higher loads than single-infection groups. For both pathogens, levels at 14 dpi were lower than at 7 dpi. Notably, the F-M group showed higher overall loads of both pathogens at 7 dpi compared to the other two co-infection groups, but lower loads at 14 dpi (Figure 6).

### 3.3 Cytokine profiles

**Figure 7:**
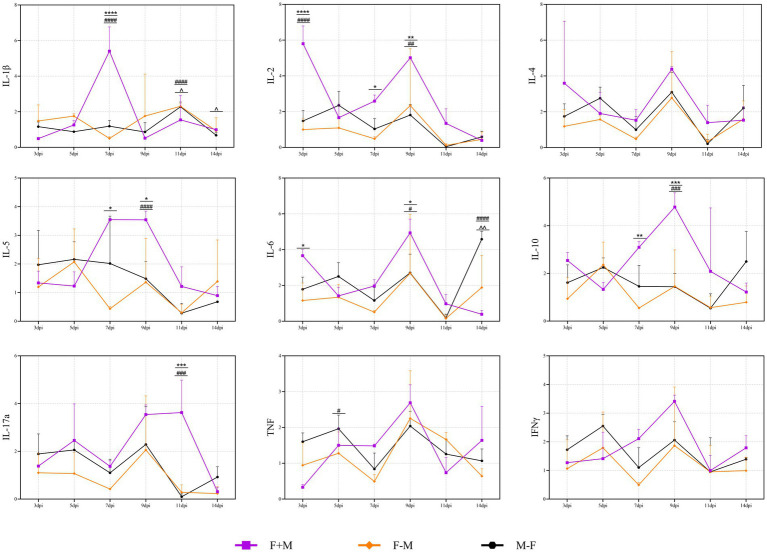
The cytokine profile changes in the co-infection group. * indicates a significant difference between the F+M group and the F-M group (*p < 0.05, **p < 0.01, ***p < 0.001, ****p < 0.0001). # indicates a significant difference between the F+M group and the M-F group (#*p* < 0.05, ##*p* < 0.01, ###*p* < 0.001, ####*p* < 0.0001). ^ indicates a significant difference between the F-M group and the M-F group (^*p* < 0.05, ^^*p* < 0.01, ^^^*p* < 0.001, ^^^^*p* < 0.0001).

The dynamic changes in the mRNA expression levels of nine key cytokines (IL-1β, IL-2, IL-4, IL-5, IL-6, IL-10, IL-17a, TNF, IFN-γ) in peripheral blood across all groups were systematically analyzed by qRT-PCR (Figure 7). The results revealed distinct cytokine response patterns that were closely associated with the temporal sequence of co-infection and infection progression.

Overall, the F+M group exhibited the most sustained and robust cytokine upregulation throughout the 14-day observation period. Most cytokines in this group peaked at 7–9 dpi, with IL-5, IL-6, IL-10, IL-17a, TNF, and IFN-γ showing the most prominent increases. In contrast, the sequential co-infection groups displayed divergent response dynamics.

The F-M group showed a “delayed peak” pattern: cytokine levels were relatively low at 3–7 dpi (IL-5 and IL-10 had one peak on 5 dpi) but surged sharply at 9 dpi (IL-1β at 11 dpi), particularly for IL-1β, IL-17a, and TNF. By 11 or 14 dpi, these pro-inflammatory cytokines rapidly declined but remained higher than the single-infection groups.

The M-F group exhibited moderate and transient cytokine elevation, with most indicators peaking at 5 dpi and 9 dpi and gradually returning to near-baseline levels by. However, most cytokines showed an slight increase again at 14 dpi, with the rebound of IL-4, IL-6, and IL-10 being more pronounced.

**Figure 8:**
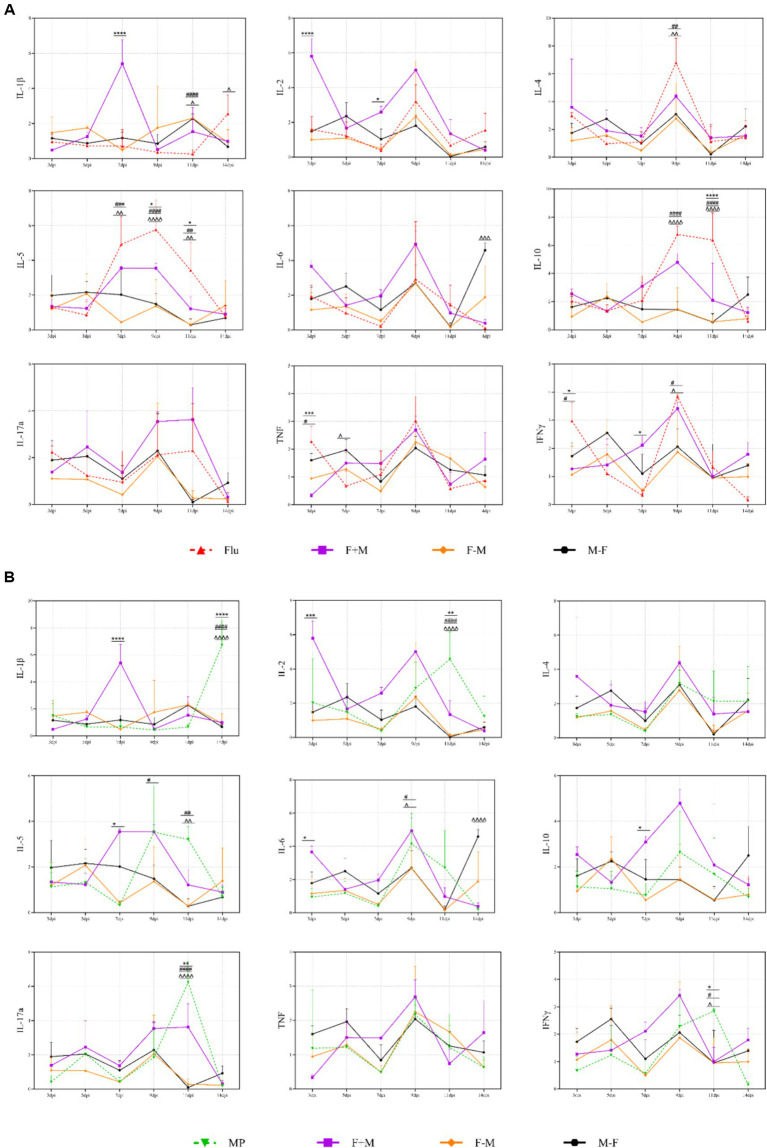
The contrast between the three co-infection groups and the Flu group or the MP group in cytokines. **(A)** The significant differences between the three co-infection groups and the Flu group. **(B)** The significant differences between the three co-infection groups and the MP group in cytokines. * indicates a significant difference between the F+M group and the Flu group or the MP group (*p < 0.05, **p < 0.01, ***p < 0.001, ****p < 0.0001). # indicates a significant difference between the F-M group and the Flu group or the MP group (#*p* < 0.05, ##*p* < 0.01, ###*p* < 0.001, ####*p* < 0.0001). ^ indicates a significant difference between the M-F group and the Flu group or the MP group (^*p* < 0.05, ^^*p* < 0.01, ^^^*p* < 0.001, ^^^^*p* < 0.0001).

Compared to the single-infection groups (Flu and MP), all co-infection groups (F+M, F-M, M-F) showed significantly enhanced cytokine responses (Figure 8). Specifically:

Relative to the Flu group, the F-M group had higher expression of IL-1β and TNF at 11 dpi (*p* < 0.001), while the F+M group showed higher IL-1β, IL-2, and IL-17a at 9–14 dpi (*p* < 0.01).

Compared to the MP group, the F+M group displayed sustained upregulation of IL-1β, IL-4, IL-6, and IL-10 (*p* < 0.05 at 7–14 dpi), reflecting a mixed Th1/Th2 response, whereas the F-M group showed selective amplification of pro-inflammatory cytokines (IL-1β and IL-17a) without corresponding increases in anti-inflammatory IL-10 (*p* < 0.01 vs. MP group at 11 dpi).

Notably, anti-inflammatory cytokine IL-10 showed a unique pattern: it was strongly correlated with pro-inflammatory cytokines (*r* = 0.837–0.862, *p* < 0.001; Supplementary Table S3) in the F+M and M-F groups, suggesting a compensatory anti-inflammatory response. However, this correlation was weakened in the F-M group (*r* = 0.383, *p* = 0.021), indicating a dysregulated balance between pro- and anti-inflammatory pathways in this group.

Pearson correlation analysis further confirmed that cytokine expression levels were significantly positively correlated with pathogen loads in nasal, pharyngeal, anal swabs, and tissues (*r* = 0.785–0.904, *p* < 0.001; Supplementary Table S3). This suggests that sustained pathogen replication directly drives the overactivation of immune cells and cytokine secretion, forming a “pathogen-cytokine positive feedback loop” in co-infection scenarios.

### 3.4 Histopathological damage

**Figure 9:**
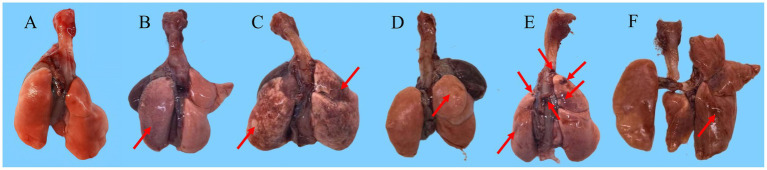
Pathological changes in lung anatomy. **(A)** Control group. **(B)** Flu group: reddish-brown tissue with uneven coloration and darker, denser areas indicating moderate infection. **(C)** MP group: patchy reddish-brown discoloration with dark regions suggesting hemorrhage, congestion, or inflammation; swollen tissue with inflammatory edema; inflammatory exudates around the hilum and prominent hemorrhagic foci indicating severe infection. **(D)** F+M group: brownish-tan tissue with minor hemorrhagic spots and slight darkening; lacking normal gloss and elasticity, consistent with mild infection. **(E)** F-M group: brownish-tan tissue with mild darkening and diminished surface luster; dark regions suggesting hemorrhage, congestion, or inflammation; inflammatory exudates and vascular congestion at the hilum with large bleeding points indicating severe infection. **(F)** M-F group: reddish-brown tissue with localized dark areas suggestive of hemorrhage or congestion; uneven and rough surface texture.

Macroscopic evaluation of lung revealed the pulmonary architecture was disrupted by the presence of punctate and patchy hemorrhages, suggesting widespread vascular injury and leakage. Furthermore, well-demarcated, consolidated lesion sites were observed, which are typically associated with extensive inflammatory cell influx, alveolar collapse, or possible fibrinous exudation. These pathological changes were accompanied by a marked alteration in the overall color and luster of the lung surface, which appeared dull and mottled with areas of pallor and hyperemia, collectively painting a picture of severe and diffuse tissue damage (Figure 9).

**Figure 10:**
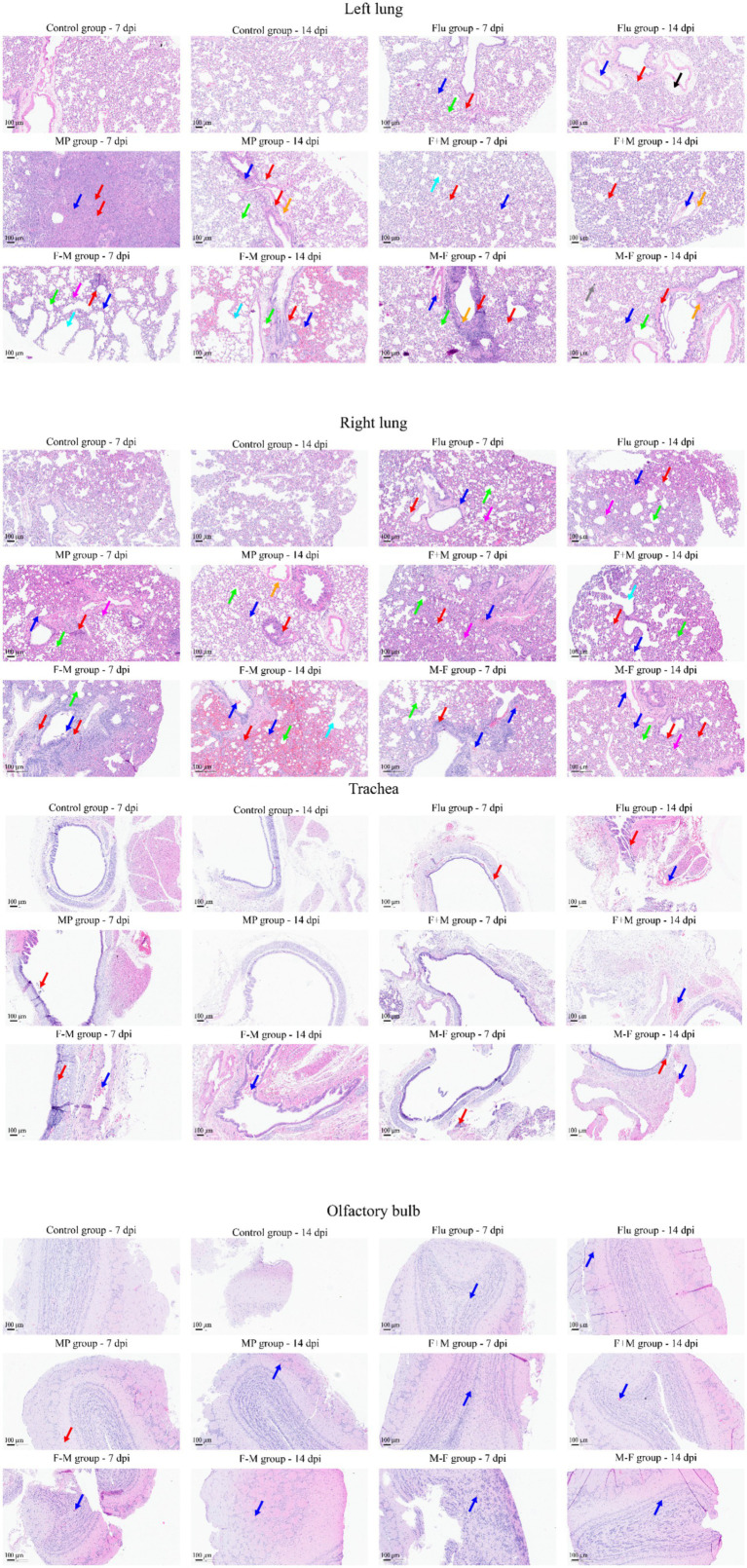
Pathological changes in respiratory tissues. Left lungs, right lungs, trachea, and olfactory bulbs were used as examples to evaluate infection-induced tissue damage. Congestion and hyperemia (blue arrows), inflammatory cell infiltration (red arrows), fibrous tissue hyperplasia (orange arrows), alveolar space dilation (green arrows), septal thickening (pink arrows), pulmonary bullae (cyan arrows), perivascular edema (black arrows), yellowish-brown pigmentation (gray arrows).

Histopathological analysis via H&E staining revealed that all infected groups exhibited partial alveolar septal thickening, mild congestion and stasis in alveolar septa, scattered inflammatory cell infiltration in bronchial epithelium, sporadic inflammatory cell infiltration, and perivascular fibrous tissue hyperplasia. The F-M group at 14 dpi displayed the most severe pathology among all groups, while the severity in other groups was relatively comparable. At 7 dpi, the lungs and olfactory bulbs of all five infected groups showed similar pathological manifestations, including alveolar septal thickening, mild-to-moderate congestion and stasis in alveolar septa, localized alveolar cavity dilation, scattered inflammatory cell infiltration, and mild congestion in the olfactory bulb. The trachea of the Flu, MP, F-M, and M-F groups exhibited mild congestion and stasis, with scattered inflammatory cell infiltration in the submucosa, while the F+M group showed no significant tracheal abnormalities. At 14 dpi, all groups showed thickening of the alveolar septa, mild to moderate congestion and hemorrhage in the alveolar septa, a few blood cells adhering to the bronchial tubes, expansion of the alveolar cavities, and scattered infiltration of inflammatory cells. The MP, F+M, and M-F groups also exhibited symptoms of fibrous tissue proliferation. In terms of lung tissue severity, the F-M group exhibited the most severe symptoms, followed by the Flu group; the MP, F+M, and M-F groups were relatively similar and milder. In the trachea, the Flu and M-F groups showed inflammatory cell infiltration, and except for the MP group, all other groups had mild congestion and hemorrhage. In the olfactory bulbs, except for the Flu group, all other groups had mild congestion and hemorrhage (Figure 10). Pathological findings in other tissues were minimal, presenting only mild congestion or inflammatory cell infiltration, with some tissues showing no notable abnormalities.

**Figure 11:**
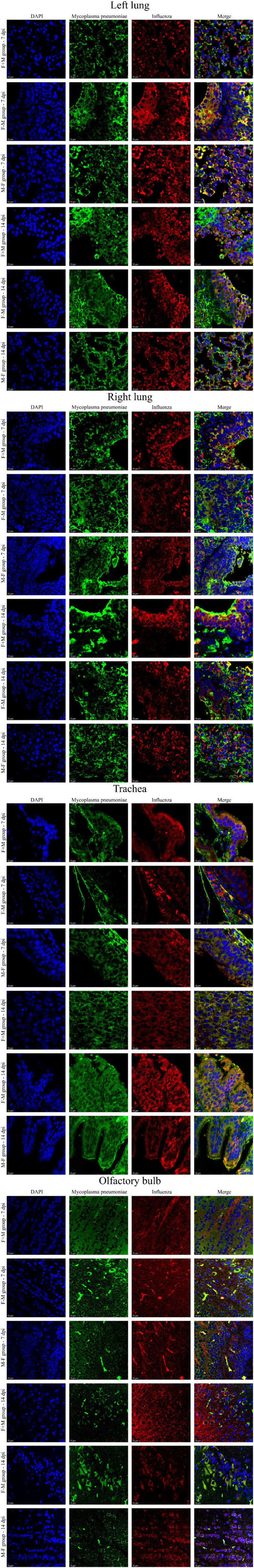
Co-localization of influenza H1N1 and *Mycoplasma pneumoniae* antigens in left lung, right lung, trachea, and olfactory bulb tissue. Green fluorescence represents the antigen protein of *Mp*, red fluorescence represents the antigen protein of influenza virus H1N1.

The IF staining of tissue section samples from animals, taking left lung, right lung, trachea and olfactory bulb tissues as examples, revealed that the co-infection group of Syrian hamsters with influenza H1N1 and *Mycoplasma pneumoniae* demonstrated co-localization of viral antigen proteins and nucleic acids. The nucleic acid signals of influenza H1N1 and MP appeared in green and red, respectively, and were distributed in sheet-like clusters around the cell nuclei (Figure 11).

## 4 Discussion

In this study, we established a Syrian hamster model of H1N1-Mp co-infection and systematically investigated his impact of infection sequence on disease severity. Our results demonstrate that the temporal sequence of co-infection is a key determinant of pathogenic outcomes, with the F-M group (H1N1 followed by Mp) exhibiting the most severe clinical symptoms, tissue damage, and immunoreaction. This finding highlights the “time window effect” in respiratory pathogen invasion—early H1N1 infection reshapes the respiratory microenvironment (e.g., epithelial barrier damage, impaired mucociliary clearance), creating an “ecological niche” for subsequent Mp colonization (Lobo et al., 2013).

### 4.1 Clinical indicator monitoring

As a result, we can observe the phenomenon that the F-M group (infection with influenza H1N1 followed by *Mycoplasma pneumoniae* 72 h later) is more severe than other groups. In clinical symptoms, we can observe that all groups developed nasal discharge and nasopharyngeal secretions and nasal bleeding was observed in the M-F group, F+M group and MP group. But there is no significantly change of body temperature and body weight (Figure 2). Studies have indicated that different strains of experimental animals exhibit variations in their body temperature responses to pathogen infection.

Changes in blood routine and biochemical parameters reflect the impact of infection on the host’s immune system and physiological functions. Hematological and cytokine analyses indicate that co-infection induces a more complex immunomodulatory pattern. In co-infected groups, WBC and CRP levels remained elevated at 14 dpi (Figure 3), with the F-M group exhibiting the highest values among all groups, suggesting persistent inflammatory responses. The persistently elevated WBC (especially the F-M group having the highest levels) and CRP at 14 dpi in co-infected groups, particularly F-M, strongly indicate ongoing and unresolved inflammation. CRP, an acute-phase protein synthesized by the liver primarily in response to IL-6, serves as a sensitive marker for systemic inflammation (Ng et al., 2018). Its sustained elevation beyond the typical acute phase (7 dpi) suggests a failure to resolve the inflammatory response effectively. In the F+M group, LYMPH% initially decreased and then increased, indicating that influenza virus infection was more severe in the early stage, while Mp infection became predominant later. The initial decrease likely reflects the early immunosuppressive or immunoreactions of simultaneous viral and bacterial assault. Influenza virus is known to induce lymphopenia early in infection, and the combined stress might transiently suppress bone marrow output or sequester cells (Sherban et al., 2023). The subsequent increase, surpassing other groups by 14 dpi, suggests delayed but sustained immune activation. This aligns with the prolonged pathogen load observed in this group (Figure 6), indicating that simultaneous infection creates a niche where both pathogens persist, driving chronic inflammation. *Mycoplasma pneumoniae*, known for its ability to establish persistent infections and evade immune clearance, likely becomes the dominant driver later, perpetuating the immune response. Influenza A virus infection is well-documented to cause transient neutropenia/lymphopenia followed by rebound (Sharon et al., 2011). The persistent decline here, indicates a more severe and sustained suppression of innate myeloid cell populations. The rise in lymphocytes (LYMPH%) likely reflects a robust adaptive immune response (T and B cells) mounting against the viral infection (Hufford et al., 2015). The statement “*Mycoplasma pneumoniae* exacerbated the influenza virus infection” is mechanistically supported by this dysregulation of innate immunity (Wu et al., 2025), creating an environment where viral pathology is amplified. According to the correlation analysis results, with the exception of PLT, all other blood indicators showed significant correlations with all cytokines (*p* < 0.05). These findings indicate that the virus caused infection in the body, led to viremia, and triggered an immune response (Supplementary Table S3).

### 4.2 Viral replication kinetics

From the swab pathogen load analysis, influenza virus can initiate replication within 6–8 h post-infection, with viral RNA detectable locally within 12 h and a significant increase within 24 h. In some animal models, transient low levels of viral RNA may be detected in blood or fecal samples within 24–48 h post-infection, which reflects early viral dissemination or swallowing rather than established systemic infection (Zheng et al., 2025; Mori et al., 1995). The F+M group peaked at 7 or 9 dpi and subsequently exhibited a secondary rise, maintaining a relatively high level thereafter. In contrast, the F-M group sustained a consistently low viral load over an extended period. The M-F group, after peaking at 7 or 9 dpi, declined rapidly, with its load remaining lower than the other two co-infected groups in later stages. These findings suggest that Mp may enhance influenza virus infection, while influenza virus appears to suppress Mp infection, aligning with subsequent tissue viral load results (Figure 5). The two pathogens may engage in direct competition (e.g., competition for receptor binding sites), resulting in a bimodal pattern in pathogen load dynamics (Figure 4), without triggering sustained inflammatory injury. This milder pathological outcome might be associated with early synchronized regulation of immune responses.

In the tissue pathogen load analysis, both pathogens were detected in all tissues, with higher loads observed in respiratory-related tissues. However, in bronchoalveolar lavage fluid and nasal lavage fluid, Mp showed a higher load, while influenza virus load was relatively lower. This discrepancy may be attributed to the fact that Mp binds to cell surface receptors without entering the cells, whereas influenza virus enters the cells for infection after receptor binding (Waites et al., 2017; Iwatsuki-Horimoto et al., 2018). Consequently, lavage fluid cannot effectively wash down a significant amount of influenza virus (Figure 6). The pathogen loads at 14 dpi were lower than those at 7 dpi, indicating gradual recovery from the disease. At 7 dpi, the F-M group exhibited the highest viral load, which, combined with pathological findings, confirmed the most severe infection in this group. Although the F-M group had a high viral load at 7 dpi, severe pathological outcomes were observed at 14 dpi (Figure 10). This suggests that the severe infection in the F-M group rapidly triggered the immune system, leading to significant tissue damage after viral reduction, highlighting that Mp may enhance influenza virus infection. This aligns with the findings of Smith et al., who demonstrated that *Streptococcus pneumoniae* promotes influenza virus infection (Smith et al., 2013). At 14 dpi, the F+M group showed a higher viral load, indicating prolonged infectivity of the co-infection. This phenomenon suggests that Mp infection might delay influenza virus clearance, consistent with the “virus-bacteria synergy in disrupting host immune responses” theory proposed by Bakaletz (2017). This parallels the cytokine profile where the F+M group maintained persistently elevated levels, while the F-M group exhibited only transient peaks at 7 or 9 dpi (Figure 7). Similarly, in influenza virus viral load detection, significant differences were observed between co-infected and single infected groups at different time points, implying that co-infection alters the replication and shedding patterns of influenza virus in the host (McCullers, 2006). According to the correlation analysis results, significant correlations were observed between cytokines and pathogen nucleic acid load (*p* < 0.05). These findings suggest that during animal infection with pathogens, viral replication directly stimulates immune cells to release cytokines, thereby triggering an immune response (Supplementary Table S3).

### 4.3 Immunoreaction and cytokine profiles

The cytokine profile data highlight that the temporal sequence of H1N1-Mp co-infection induces distinct levels of immunoreaction, which are critical drivers of disease severity—consistent with the “cytokine storm” hypothesis in respiratory co-infections (Lalbiaktluangi et al., 2023; Thomas et al., 2009).

The sustained and global cytokine upregulation in the F+M group (Figure 7) is likely a result of synergistic immune activation by both pathogens. Influenza H1N1 activates the NLRP3 inflammasome via its M2 ion channel, triggering IL-1β and TNF secretion (Ichinohe et al., 2010), while Mp enhances pro-inflammatory responses through its lipid-associated membrane proteins (LAMPs) and CARDS toxin (Atkinson et al., 2008; Medina et al., 2017). Simultaneous exposure to these stimuli disrupts the host’s immune homeostasis, leading to prolonged production of Th1 (IFN-γ, IL-2), Th2 (IL-4, IL-5), and Th17 (IL-17a) cytokines. The persistent elevation of IL-6 and CRP (Figure 3) in this group further supports ongoing systemic inflammation, as IL-6 is a key inducer of hepatic CRP synthesis (Ng et al., 2018). This mixed immune response may explain the prolonged pathogen load and mild-to-moderate tissue damage observed in the F+M group, as Mp’s ability to establish persistent infections (Waites et al., 2017) perpetuates immune activation even as H1N1 replication declines.

In contrast, the F-M group exhibited a phenomenon of delayed pro-inflammatory surge (Figure 7). Prior H1N1 infection disrupts the respiratory epithelial barrier (Krammer et al., 2018) and impairs mucociliary clearance, creating a permissive microenvironment for subsequent Mp colonization. This sequential exposure may prime immune cells (e.g., macrophages, neutrophils) via TLR2 activation by Mp’s LAMPs (Atkinson et al., 2008), leading to a delayed but explosive release of pro-inflammatory cytokines at 11 dpi. Notably, the lack of a corresponding IL-10 elevation in this group (Figure 7) suggests a failure of the anti-inflammatory feedback mechanism, which is consistent with the most severe histopathological damage observed in the F-M group (Figure 10). This finding aligns with Smith et al.’s observation that sequential viral-bacterial infection amplifies pro-inflammatory responses by disrupting innate immune regulation (Smith et al., 2013) ultimately exacerbating tissue injury.

The M-F group’s transient and moderate cytokine response indicates that prior Mp infection may attenuate H1N1-induced immune overactivation. Mp is known to modulate host immune responses through the secretion of immunomodulatory factors (Chaudhry et al., 2016), which may limit the magnitude of H1N1-triggered cytokine production. The rapid decline in cytokine levels after 7 dpi or 9 dpi (Figure 7) correlates with the early clearance of both pathogens in this group (Figure 4), supporting the notion that Mp-first infection reduces the synergistic pathogenicity of the two pathogens.

Although the F+M group showed the highest cytokine peaks at certain time points, the F–M group displayed features consistent with immune dysregulation, characterized by persistent pro-inflammatory cytokine expression at later stages, weakened pro-inflammatory balance, and the most severe lung pathology despite moderate cytokine peaks. These findings suggest that tissue damage is more closely associated with dysregulated immune responses than with the absolute magnitude of cytokine production.

Furthermore, the correlation analysis (Supplementary Table S3) confirms that cytokine levels are tightly linked to pathogen loads across all groups, emphasizing that persistent pathogen replication is a key driver of immunoreaction. This is consistent with Mifsud et al.’s report that influenza virus replication in the upper respiratory tract directly stimulates innate immune cells to secrete pro-inflammatory cytokines (Mifsud et al., 2021). In co-infection, the combined replication of H1N1 and Mp amplifies this effect, leading to more strong immunoreaction than single infections—particularly in the F-M group, where H1N1-induced epithelial damage enhances Mp colonization and sustained immune stimulation.

Collectively, these findings demonstrate that the temporal sequence of H1N1-Mp co-infection dictates the nature and magnitude of cytokine responses: simultaneous infection induces sustained, mixed pro/anti-inflammatory responses, while H1N1-first infection triggers a delayed, unbalanced pro-inflammatory surge. These distinct immunoreaction levels underpin the differences in disease severity observed across groups, highlighting the critical role of cytokine networks in mediating the pathogenic outcomes of respiratory co-infections (Bakaletz, 2017).

### 4.4 Pathological alterations and tissue tropism

From the most direct histopathological results, most co-infection groups exhibited distinct pulmonary lesion characteristics such as pulmonary bullae, alveolar cavity dilation, and perivascular fibrosis, suggesting that co-infection may lead to impaired lung tissue repair, potentially associated with synergistic damage mechanisms between the two pathogens. *Mycoplasma pneumoniae* mediating chronic inflammatory responses via its adhesin P1 protein (Atkinson et al., 2008), while influenza virus damages respiratory ciliated epithelium via neuraminidase (Krammer et al., 2018), potentially exposing the basal membrane and facilitating *Mycoplasma pneumoniae* adhesion and colonization. Influenza virus infection was characterized by thickened alveolar septa and infiltration of inflammatory cells, while *Mycoplasma pneumoniae* infection presented with bronchitis (Figure 10). In terms of severity, the F-M group showed more severe lesions compared to the F+M and M-F groups, further supporting that *Mycoplasma pneumoniae* suppresses influenza virus infection, while influenza virus may promote *Mycoplasma pneumoniae* colonization. Conversely, *Mycoplasma pneumoniae* releases pro-inflammatory factors such as the CARDS toxin (Chaudhry et al., 2016; Medina et al., 2017), exacerbating influenza virus-induced local inflammation and causing alveolar structural damage. Following influenza virus infection, the virus first invades host cells and rapidly activates host type I interferons and inflammatory cytokines, which may indirectly inhibit *Mycoplasma pneumoniae* colonization or replication (Mifsud et al., 2021). Cytokine results showed that, except for IL-1β, interferon and inflammatory cytokine levels in the F-M group were lower than those in the other co-infection groups at 7 dpi (Figure 7). The F-M group exhibited significant peaks in pro-inflammatory cytokines such as IL-1β, IL-6, and TNF at the late stage of infection (11 dpi) (Figure 7), suggesting that prior influenza infection may enhance the excessive inflammatory response triggered by *Mycoplasma pneumoniae* via activating pathways like the NLRP3 inflammasome (Ichinohe et al., 2010; Thomas et al., 2009). When Mycoplasma subsequently invades, the activation of TLR2 by its lipid-associated membrane proteins (LAMPs) may trigger a “cytokine storm” (Atkinson et al., 2008).

### 4.5 Clinical implications and model validity

In this study, we use influenza H1N1 and *Mycoplasma pneumoniae* to infect Syrian hamsters in different temporal sequences. The findings of this research significantly enhanced the comprehension of influenza H1N1 and *Mycoplasma pneumoniae* infection in animal bodies. This finding provided important experimental evidence for the temporally dependent pathological mechanisms underlying clinical respiratory co-infections and lay the foundation for research into viral mechanisms and the development of drugs and vaccines.

Syrian hamsters are increasingly recognized as a robust model for human respiratory infections due to their similarity to humans in viral replication kinetics and clinical symptoms (Iwatsuki-Horimoto et al., 2018). Our model recapitulates key features of human co-infections, such as increased white blood cell counts and CRP levels, validating its relevance for translational research. Clinically, these findings emphasize the need for early diagnosis and targeted interventions in patients with suspected sequential H1N1-MP infections, particularly in pediatric, elderly and immunocompromised populations where co-infections are prevalent.

### 4.6 Limitations and future directions

This study used single-sex (female) hamsters and did not evaluate the potential effects of sex differences on co-infection. Additionally, only three animals were used per group, and the relatively small sample size may influence the generalizability of quantitative estimates. The infection interval was fixed at 72 h, and further studies may help optimize the timing window. A limitation of this study is the lack of a histopathological scoring system for the quantitative assessment of lung injury. The direct interaction between Mp and the influenza virus at the cellular level (such as the regulation of TLR signaling pathways by co-infection) was also beyond the scope of the current study. Future studies are recommended to use organoid models or single-cell sequencing techniques to explore these interactions in depth. It is necessary to test temporally dependent intervention strategies (such as the preventive use of MP antibiotics after influenza infection) for their effectiveness in the model.

In conclusion, this study demonstrates that the temporal sequence of H1N1 and MP co-infection profoundly influences pathogenesis in Syrian hamsters. The enhanced severity observed in the F-M group underscores the need for further research into the molecular mechanisms underlying sequential respiratory infections and highlights the utility of this animal model for preclinical evaluations of antiviral and antibacterial interventions.

## Data Availability

The original contributions presented in the study are included in the article/Supplementary material, further inquiries can be directed to the corresponding authors.
