# Supplementary material for: The temporal sequence of influenza H1N1 and Mycoplasma pneumoniae co-infection causes disease severity in Syrian hamster models
Source: Front Microbiol. 2026 Mar 27;17:1787294. doi: 10.3389/fmicb.2026.1787294 (PMC13066289; doi:10.3389/fmicb.2026.1787294)
Supplement: Supplementary file 1 [file Table_1.docx]

**Supplementary Material**

**Table S1.** The primers and probes of influenza H1N1 and Mycoplasma pneumoniae:

| Primers | Sequences |
| --- | --- |
| FluA-Forward | GACCAATCCTGTCACCTCTGAC |
| FluA-Reverse | GGGCATTTTGGACAAAGCGTCTACG |
| FluA-Probe | TGCAGTCCTCGCTCACTGGGCACG |
| P1a-F | GGCGTTTGCGGGTTTAACTT |
| P1a-R | CTTTGCGTTACGCCGGTATG |
| P1a-Probe | CGCCGGGCGCGCCTTATACG |
