# Supplementary material for: The temporal sequence of influenza H1N1 and Mycoplasma pneumoniae co-infection causes disease severity in Syrian hamster models
Source: Front Microbiol. 2026 Mar 27;17:1787294. doi: 10.3389/fmicb.2026.1787294 (PMC13066289; doi:10.3389/fmicb.2026.1787294)
Supplement: Supplementary file 2 [file Table_2.docx]

**Supplementary Material**

**Table S2.** The primers of cytokines:

| Primers | Sequences |
| --- | --- |
| IL1β-F | AACAGCGGCCAGGATTTAG |
| IL1β-R | TCTTCGTGTGTCAGCATTAGG |
| IL2-F | GTGGTGGAATTTCTGAACAGATG |
| IL2-R | GTGTTGTAGGAGGCAGGTAAT |
| IL4-F | GAAGAACTCCACGGAGAAAGAC |
| IL4-R | GGGTCACCTCATGTTGGAAATA |
| IL5-F | CTCAATTCCCATGAGGCTAACT |
| IL5-R | CTACTCCAGGCACAGAAAGATG |
| IL6-F | GGGATGTCTGTAGCTCATTCTG |
| IL6-R | CTTGCGGAGAGGAACTTCATAG |
| IL10-F | CATTTCACAGCTACAGACGTTTAC |
| IL10-R | CCTTCCTAAGCCCTGTCTTTAC |
| IL17a-F | AGCTCAGGATGTCCAAACAC |
| IL17a-R | GGCCTTCTGGAACTCACTTT |
| TNF-F | GACTACAGAACACCCTGGAAATAG |
| TNF-R | TGTGTACGAGAGGGACAGAA |
| IFN-γ-F | GCCAGATCGTCTCCTTCTACT |
| IFN-γ-R | GTCTGCCTTGATGGTGTCTATG |
| PPIA-F | GCGTCTCCTTCGAGCTATTT |
| PPIA-R | CTGTGAAAGGAGGAACCCTTAT |
| HPRT1-F | GATCCATTCCCATGACTGTAGAT |
| HPRT1-R | GAGAGATCGTCTCCACCAATAAC |
