# Supplementary material for: The temporal sequence of influenza H1N1 and Mycoplasma pneumoniae co-infection causes disease severity in Syrian hamster models
Source: Front Microbiol. 2026 Mar 27;17:1787294. doi: 10.3389/fmicb.2026.1787294 (PMC13066289; doi:10.3389/fmicb.2026.1787294)
Supplement: Supplementary file 3 [file Table_3.docx]

**Supplementary Material**

**Table S3.** The correlation among blood parameters, pathogen load, and cytokine levels

|  | | IL1β | IL2 | IL4 | IL5 | IL6 | IL10 | IL17a | TNF | IFNγ |
| --- | --- | --- | --- | --- | --- | --- | --- | --- | --- | --- |
| WBC | Pearson correlation | 0.076 | 0.087 | 0.1 | 0.089 | 0.079 | 0.062 | 0.109 | 0.09 | 0.111 |
|  | Significance (dual tailed) | 0.661 | 0.615 | 0.562 | 0.605 | 0.648 | 0.72 | 0.527 | 0.602 | 0.519 |
|  | Number of cases | 36 | 36 | 36 | 36 | 36 | 36 | 36 | 36 | 36 |
| PLT | Pearson correlation | -0.294 | -0.297 | -0.282 | -0.296 | -0.298 | -0.308 | -0.256 | -0.267 | -0.266 |
|  | Significance (dual tailed) | 0.082 | 0.079 | 0.096 | 0.08 | 0.078 | 0.068 | 0.132 | 0.116 | 0.116 |
|  | Number of cases | 36 | 36 | 36 | 36 | 36 | 36 | 36 | 36 | 36 |
| LYMPH | Pearson correlation | 0.317 | .382^*^ | .405^*^ | .350^*^ | .418^*^ | .383^*^ | .389^*^ | .342^*^ | .406^*^ |
|  | Significance (dual tailed) | 0.06 | 0.022 | 0.014 | 0.036 | 0.011 | 0.021 | 0.019 | 0.041 | 0.014 |
|  | Number of cases | 36 | 36 | 36 | 36 | 36 | 36 | 36 | 36 | 36 |
| CRP | Pearson correlation | .575^**^ | .611^**^ | .609^**^ | .609^**^ | .615^**^ | .609^**^ | .627^**^ | .614^**^ | .642^**^ |
|  | Significance (dual tailed) | 0 | 0 | 0 | 0 | 0 | 0 | 0 | 0 | 0 |
|  | Number of cases | 36 | 36 | 36 | 36 | 36 | 36 | 36 | 36 | 36 |
| NasalSwabDNA | Pearson correlation | .825^**^ | .867^**^ | .814^**^ | .800^**^ | .872^**^ | .846^**^ | .830^**^ | .792^**^ | .792^**^ |
|  | Significance (dual tailed) | 0 | 0 | 0 | 0 | 0 | 0 | 0 | 0 | 0 |
|  | Number of cases | 88 | 90 | 90 | 86 | 90 | 90 | 90 | 90 | 90 |
| ThroatSwbaDNA | Pearson correlation | .836^**^ | .875^**^ | .824^**^ | .804^**^ | .877^**^ | .856^**^ | .838^**^ | .798^**^ | .796^**^ |
|  | Significance (dual tailed) | 0 | 0 | 0 | 0 | 0 | 0 | 0 | 0 | 0 |
|  | Number of cases | 88 | 90 | 90 | 86 | 90 | 90 | 90 | 90 | 90 |
| AnalSwabDNA | Pearson correlation | .833^**^ | .871^**^ | .816^**^ | .806^**^ | .874^**^ | .850^**^ | .833^**^ | .795^**^ | .795^**^ |
|  | Significance (dual tailed) | 0 | 0 | 0 | 0 | 0 | 0 | 0 | 0 | 0 |
|  | Number of cases | 88 | 90 | 90 | 86 | 90 | 90 | 90 | 90 | 90 |
| BloodDNA | Pearson correlation | .648^**^ | .770^**^ | .769^**^ | .802^**^ | .808^**^ | .755^**^ | .800^**^ | .855^**^ | .878^**^ |
|  | Significance (dual tailed) | 0 | 0 | 0 | 0 | 0 | 0 | 0 | 0 | 0 |
|  | Number of cases | 88 | 90 | 90 | 86 | 90 | 90 | 90 | 90 | 90 |
| NasalSwabRNA | Pearson correlation | .815^**^ | .860^**^ | .804^**^ | .795^**^ | .866^**^ | .837^**^ | .822^**^ | .786^**^ | .788^**^ |
|  | Significance (dual tailed) | 0 | 0 | 0 | 0 | 0 | 0 | 0 | 0 | 0 |
|  | Number of cases | 88 | 90 | 90 | 86 | 90 | 90 | 90 | 90 | 90 |
| ThroatSwabRNA | Pearson correlation | .816^**^ | .861^**^ | .805^**^ | .797^**^ | .866^**^ | .838^**^ | .823^**^ | .787^**^ | .789^**^ |
|  | Significance (dual tailed) | 0 | 0 | 0 | 0 | 0 | 0 | 0 | 0 | 0 |
|  | Number of cases | 88 | 90 | 90 | 86 | 90 | 90 | 90 | 90 | 90 |
| AnalSwabRNA | Pearson correlation | .813^**^ | .859^**^ | .802^**^ | .796^**^ | .865^**^ | .835^**^ | .821^**^ | .785^**^ | .788^**^ |
|  | Significance (dual tailed) | 0 | 0 | 0 | 0 | 0 | 0 | 0 | 0 | 0 |
|  | Number of cases | 88 | 90 | 90 | 86 | 90 | 90 | 90 | 90 | 90 |
| BloodRNA | Pearson correlation | .623^**^ | .746^**^ | .748^**^ | .781^**^ | .786^**^ | .732^**^ | .779^**^ | .837^**^ | .860^**^ |
|  | Significance (dual tailed) | 0 | 0 | 0 | 0 | 0 | 0 | 0 | 0 | 0 |
|  | Number of cases | 88 | 90 | 90 | 86 | 90 | 90 | 90 | 90 | 90 |
| tissue7dpiDNA | Pearson correlation | .680^**^ | .806^**^ | .781^**^ | .826^**^ | .825^**^ | .779^**^ | .820^**^ | .842^**^ | .873^**^ |
|  | Significance (dual tailed) | 0 | 0 | 0 | 0 | 0 | 0 | 0 | 0 | 0 |
|  | Number of cases | 88 | 90 | 90 | 86 | 90 | 90 | 90 | 90 | 90 |
| tissur14dpiDNA | Pearson correlation | .684^**^ | .810^**^ | .785^**^ | .829^**^ | .829^**^ | .783^**^ | .824^**^ | .845^**^ | .876^**^ |
|  | Significance (dual tailed) | 0 | 0 | 0 | 0 | 0 | 0 | 0 | 0 | 0 |
|  | Number of cases | 88 | 90 | 90 | 86 | 90 | 90 | 90 | 90 | 90 |
| tissue7dpiRNA | Pearson correlation | .725^**^ | .847^**^ | .820^**^ | .860^**^ | .862^**^ | .821^**^ | .857^**^ | .870^**^ | .904^**^ |
|  | Significance (dual tailed) | 0 | 0 | 0 | 0 | 0 | 0 | 0 | 0 | 0 |
|  | Number of cases | 88 | 90 | 90 | 86 | 90 | 90 | 90 | 90 | 90 |
| tissue14dpiRNA | Pearson correlation | .724^**^ | .845^**^ | .816^**^ | .860^**^ | .862^**^ | .818^**^ | .855^**^ | .870^**^ | .901^**^ |
|  | Significance (dual tailed) | 0 | 0 | 0 | 0 | 0 | 0 | 0 | 0 | 0 |
|  | Number of cases | 88 | 90 | 90 | 86 | 90 | 90 | 90 | 90 | 90 |
